# Nurses’ perceptions regarding the establishment of clinical education and training units in public hospitals in the North-West Province: a qualitative study

**DOI:** 10.1186/s12912-024-02449-z

**Published:** 2024-10-25

**Authors:** Mmapule Motshabi Maria Mosete, Isaac Ontchebile Mokgaola, Leepile Alfred Sehularo, Miriam Mmamphamo Moagi

**Affiliations:** 1 NuMIQ Research Entity, Faculty of Health Sciences, North-West University, Mahikeng , South Africa; 2 Lifestyle Diseases Research Entity, Faculty of Health Sciences, North-West University, Mahikeng, South Africa; 3 Nursing Department, Sovenga, University of Limpopo, Polokwane, Limpopo Province South Africa

**Keywords:** Clinical education, Clinical training, Clinical unit, Establishment, Perception, Professional nurses

## Abstract

**Background:**

A Clinical Education and Training Unit is defined as a unit in which the clinical orientation, teaching, learning, and assessment, mentoring and support of student nurses are coordinated to achieve the outcomes of their learning programmes in the health establishment. In South Africa, Clinical Education and Training Unit was developed to reconstruct and revitalise nursing education and training. The clinical education is crucial to enable nursing students to put what they learn in the classroom into practice as they direct patient care, practise therapeutic communication, hone technical skills, demonstrate caring behaviours, investigate ethical dilemmas, and take on the roles of nurses. The clinical teaching unit is one of the models that show potential in advancing clinical education. Since the proposed clinical education and teaching model concluded that Clinical Education and Training Units should be established in 2012, to the researcher’s knowledge, no research study has been conducted to explore and describe the nurses’ perceptions regarding the establishment of Clinical Education and Training Units, particularly in the North-West Province. This has resulted in the scarcity of literature on their perceptions. Thus, the researcher believed that it was essential to explore and describe the perceptions of professional nurses regarding the establishment of Clinical Education and Training Units in the North-West Province of South Africa, so that their perceptions may be known and understood.

**Aim:**

The study aimed to explore and describe the perceptions of professional nurses regarding the establishment of Clinical Education and Training Units in public hospitals in the North-West Province.

**Methods:**

A qualitative-exploratory-descriptive and contextual research design was used. This method was used because it is known to unearth a wide range of views, opinions, thoughts, and feelings of the participants thus enabling a deeper understanding concerning perceptions of professional nurses regarding the establishment of Clinical Education and Training Units at the hospitals of interest. Participants were recruited using pamphlets and flyers. A non-probability convenience and purposive sampling techniques were used to select participants based on an inclusion criterion. From 09 September 2023 to 07 December 2023, the researcher used face-to-face group discussions to collect data and it was analysed using six steps of inductive thematic data analysis as outlined by Braun and Clarke.

**Results:**

Four focus groups discussions were conducted with a total of 19 participants who were professional nurses. Responses were categorised into three domains: advantages of the Clinical Education and Training Unit, challenges of the Clinical Education and Training Unit, and recommendations for the Clinical Education and Training Unit. Subsequently, a total of three main themes with eight sub-themes were determined for advantages of the Clinical Education and Training Unit, two themes with six subthemes for challenges of the Clinical Education and Training Unit, and one theme with two sub-themes for recommendations for the Clinical Education and Training Unit respectively.

**Conclusion:**

The need to establish functional Clinical Education and Training Units is therefore very important not only to student nurses but also to health care professionals. Most of the participants emphasised that if Clinical Education and Training Units were fully established they would be beneficial in developing health care professionals, especially in terms of in-service training where nursing standards are emphasised as outlined by the South African Nursing Council. The research study results might contribute to the full establishment of CETUs in the hospitals of the North- West Province, South Africa.

**Trial registration:**

not applicable to the study.

## Introduction

Clinical education is essential in nursing education and training as it develops student nurses professionally [1]. Furthermore, clinical education promotes a welcoming and encouraging environment to students [2]. Clinical and Training Units (CTUs) are known as Clinical Education and Training Units (CETUs) in South Africa (SA). According to South Africa Department of Health (The National Strategic Plan for Nurse Education: Training and Practice), CETU is a unit within a healthcare facility in which clinical orientation, teaching, learning, assessment, mentoring, and support for student nurses are coordinated to facilitate the achievement of clinical goals of their educational programs [3].

Ambasta et al., stated that, the Clinical and Training Unit (CTU) is a model of clinical training that is based in hospitals and uses a variety of clinical and professional skills to help students in the health sciences meet their clinical education requirements in the field of nursing [4]. This CTU is a model that was approved by the Association of Canadian Medical Colleges (ACMC) in 1962 and is designed to foster a supportive environment [5].

## Background and purpose

Historically, the Clinical Teaching Unit (CTU) model is a Canadian concept that has served Canadian Academic Medicine well for over thirty years [6]. According to Tang et al., CTU was first approved by the Association of Canadian Medical Colleges (ACMC) in 1962 [7]. CTU was firstly witnessed in Canada in 1962, where a hospital structure was used for both health and education purposes [8]. The hospitals and health education institutions jointly appointed professional nurses as teaching staff and used a team approach that involved health science students at all levels with graded responsibilities under direct supervision to render patient care [8].

Hamoen and Daniels identified the presence of dedicated, competent clinical educators as the main features of CTU [5]. The clinical educators must have good teaching skills in order to teach effectively, facilitate learning by using their knowledge and possess clinical reasoning skills to teach students and involve them in critical reasoning processes [5]. Hoffman and Daniels assert that incompetent clinical educators can negatively affect clinical teaching, resulting in poor clinical teaching and inadequate integration of theory and practice and ultimately negatively impacting patient health outcomes [9].

A research study conducted by Tang et al., stated that the overall objectives of CTU is to use patient care as a model for research and clinical education. In the light of these objectives, the proposed core principles of CTU include residency training providing an educational experience which simulates real-world practice wherever possible, creating an exemplary standard of care for both patients and broader community, and fostering a favourable environment for scientific endeavour [7].

In nursing education, CTU allows student nurses to integrate theory and practice, meaning the classroom and clinical environments are linked when students practise what they have learned in the classroom [10]. The same author added that integrating theory and practice improves student nurses’ knowledge and skills, and increases their self-confidence, thus promoting quality patient care [10]. Considering the benefits of CTU, it can be argued that CTU is crucial for educating and training student nurses. From the above information, the researcher is of the opinion that, not having CTU at a hospital that offers a practical component to student nurses might have detrimental implications on training such as the limited time for clinical education.

In the South African context, CTU was previously known as Clinical Teaching Department (CTD) which is now addressed as Clinical Education and Training Unit (CETU), concentrating only on nursing education and training. According to National guidelines for nursing clinical education and training units in South Africa, CETU is a unit in the health establishment where clinical orientation, teaching, learning, assessment, mentoring and support of student nurses are coordinated to achieve the outcomes of their learning programmes [11]. The concept of CETU was hatched after recommendations by National Department of Health Ministerial Task Team (MTT) which was later developed into a National Strategic Plan (NSP) of the Department of Health (DoH) of 2012/13 to 2016/17 for nursing education, training and practice. The NSP aims to reconstruct and revitalise nursing education and training [3].

The Ministerial Task Team (MTT) was appointed following the nursing summit hosted by the National Department of Health (NDoH) from the 5th to 7th of April 2011 at Sandton Convention Centre in Johannesburg aiming to reconstruct and revitalise nursing. Among other things, the MTT of the NDoH recommended that a devoted structure should be in place to implement the proposed clinical education and training model [3]. One of the key subjects within the proposed clinical education and training model is the establishment of CETU at hospitals and Primary Health Care (PHC) facilities approved by South African Nursing Council (SANC) for placement of student nurses for their clinical learning. CETU is primarily responsible for clinical training of student nurses to promote the quality of practical hands-on training, the staff development and in-service of qualified nursing staff and lastly, the induction and orientation of new nursing staff and community service nurses [3].

The study conducted in SA by Mtshali and Zwane, reveals that the history of formal nursing training dates as far back as the era of colonial missionaries in the 1880s, with the nurses undergoing on-the-job training that focused on the gaining of technical skills [12]. Later, nursing training became hospital-based under hospitals’ administration. The same authors further indicate that among other things, the hospital’s administration ensured that there were enough theoretical classes and CTDs supervised by professional nurses as clinical instructors [12]. The primary purpose of CTDs was to help student nurses integrate theory with practice. In the 1940s, nursing education relocated from hospital buildings to stand-alone nursing colleges and university nursing schools commonly known as Nursing Education Institutions (NEIs), a move that saw the discontinuation of CTDs [12]. Although the CTD was discontinued, the NEIs are, however, still associated with hospitals because they serve as their clinical facilities.

Although the South Africa Department of Health (The National Strategic Plan for Nurse Education: Training and Practice) pronounced that the establishment process of CETUs must be completed between 2012 and 2017 nationwide, the researcher observed that, even though the process of establishing CETUs at hospitals of interest has already been initiated since 2016, CETUs are neither fully established nor fully operational. However, to the researcher’s knowledge, no research study has been conducted to explore and describe the nurses’ perceptions regarding the establishment of CETUs, particularly in the NWP [3]. This has resulted in the scarcity of literature on their perceptions. Thus, the researcher believes that it is essential to explore and describe the perceptions of professional nurses regarding the establishment of CETUs in the NWP of South Africa, so that their perceptions may be known and understood. These hospitals receive referrals from local hospitals, community health centres, and clinics within their Districts. Furthermore, the public hospitals of interest accommodate student nurses from public and private colleges and universities in the North-West Province (NWP) to complete their practical component as per SANC requirements.

According to the guideline of CETU, CETU is a prerogative of the clinical facilities alone and it will also assist hospitals with the in-service of the staff [11]. Therefore, CETU will not only be beneficial to student nurses but also to the clinical staff. Additionally, the research study might contribute to the repository of knowledge in the South African context and close the literature gap by possibly detailing some of the undocumented information on previous practice of CTD and current practice of CETU of clinical teaching. Furthermore, the perceptions of professional nurses could help to improve the process of establishing CETUs in their hospitals, thus becoming fully functional.

## Methods

### Design and participants

The researcher used qualitative-explorative and descriptive research design to explore and describe the perceptions of professional nurses regarding the establishment of CETUs in four public hospitals in four districts of the NWP. The qualitative-explorative and descriptive research design was used to uncover a wide range of views, opinions, thoughts, and feelings of the participants thus enabling a deeper understanding of the perceptions of professional nurses regarding the establishment of CETUs at hospitals of interest [13].

Participants were recruited from a public hospital, in each district of the NWP, which are Bojanala, Dr Ruth Segomotsi Mompati, Dr Kenneth Kaunda, and Ngaka Modiri Molema. The NWP consist of four district with atleast one major public hospital where the CETUs have been established but have not yet fully functional as expected in each of all four hospitals. The decision to select one public hospital per district was mainly influenced by insuring and enhancing representation from these districts. The inclusion criteria included for example, professional nurses registered with the South African Nursing Council (SANC) and had more than two years of working experience in the applicable hospitals as they were orientated about CETUs and the researcher is of the opinion that they have more insight about CETU and will therefore be able to provide richer data.

Participants were recruited through convenience and purposive sampling techniques. Convenience sampling was used because it simply enrolled participants who are most conveniently available whereas purposive sampling was relevant for this study because participants were chosen based on their knowledge about the phenomenon of interest. Consequently, the researcher minimized cost and saved time. In this study, participants were selected based on their availability and knowledge about the phenomenon at hand. Therefore, the researcher selected participants by choice that is, participants that were readily available to participate in the study and were knowledgeable about CETU. This means the professional nurses were chosen based on their availability and knowledge of CETU. Flyers containing the content on how the research will be conducted was placed on notice boards of participating hospitals and helped to recruit professional nurses to take part on the research study.

### Data collection

Data were collected from 09 September 2023 to 07 December 2023, using face-to-face Focus Group Discussions (FGDs) with professional nurses who signed a consent form and were willing to participate in the study. In order to obtain data during FGDs, clear semi-structured open-ended questions were formulated in such a way that in-depth description of data was attained from the participants. The questions that were asked are as follows:

- What are your perceptions as professional nurses regarding the establishment of CETU in this hospital?
- What do you think can be done to fully complete the process of the establishment of CETU in this hospital?

Furthermore, clarifying, probing, follow-up questions and other communication techniques were used to expand the discussion and generate more dialogue among participants. An interview guide (a data collection tool) was tested via pilot test and the results led to no modification in the final tool. Subsequently, Four FDG consisting of four to five participants were conducted. The number of participants per FDG was mainly influenced by unavailability of participants. In contrary, the researcher is of the view that the number of participants per FDG did not affect the practical significance of the study findings. Nevertheless, the ultimate sample size was determined by data saturation. The duration of each FGD lasted atleast forty five (45) minutes to an hour. Data saturation was reached when participants were not bringing new information. The researcher clarified the FGDs’ aims to the participants and requested permission to record the discussions. An audio recorder was used, and participants were informed about the recording at the beginning of each session. Participants were reminded to remain anonymous throughout the study by not mentioning any names, but numbers used to identify participants.

### Ensuring rigour

Trustworthiness was reflected throughout the study through the following principles: Credibility, Dependability, Confirmability, and Transferability. Credibility was achieved through peer debriefing where the researcher met with qualified peers and supervisors to discuss the proposed topic and research process. Additionally, the strategy of prolonged engagement during face-to-face FGDs with professional nurses until data saturation was reached used to maintain credibility. The professional nurses were allowed to ask questions about the research topic, to think well about the matter at hand, and were given enough time to express their perceptions, thoughts, and feelings regarding the establishment of CETUs at the chosen hospitals until data saturation was reached. Dependability was attained by strictly adhering to the proposed research methodology of the study, step by step. Subsequently, dependability was attained by involving an independent researcher in co-coding and conducting audit trials. Confirmability was reached using field notes, FGDs report, and recorded data confirming the neutrality of data.

To ensure transferability, data were transcribed verbatim, meaning the recordings were fully typed out and the transcripts were accurate, reflecting the focus group discussion. Participants’ words are most important when it comes to transcribing, therefore the researcher needed to revisit the tape, read and re-read the transcribed data to make meaning out of them and record initial ideas. To ensure fairness, openness, and transparency, flyers that promoted the probability of taking part in the research study were displayed on the information boards of the hospitals of interest to attract nurses. Professional nurses voluntarily agreed to participate in the research study because participation was purely voluntary. These was to safeguard the principle of justice in qualitative research.

### Ethical considerations

The researcher obtained conditional ethical approval for the study from the North-West University-Health Research Ethics Committee (NWU-HREC) on 14/06/2023, on 21/07/2024 received approval from NWDoH, and received final approval for the study on 07/08/2023 from the North-West University-Health Research Ethics Committee (NWU-HREC) (Reference Number: NWU-00052-23-S1). Goodwill permission letters were received from the Chief Executive Officers (CEOs) of selected hospitals prior to data collection to uphold the ethical principles of research. The aim of the study was explained to participants prior to data collection and valid written informed consent from all participants was obtained prior to the collection process to maintain the trustworthiness of the study. Anonymity and confidentiality were continuously guaranteed and protected throughout the study. All data were managed according to Protection of Personal Information (POPI) Act [14]. POPI Act is an act that regulate how information of individuals must be kept to maintain privacy.

### Data analysis

The researchers and co-coder analysed data independently using Braun and Clarke’s six steps of thematic analysis [15]. These steps include, Familiarisation with data, Generation of initial codes, Searching for themes, Review of themes, Defining and naming themes and Writing the report. The step “familiarisation with data” is whereby the researcher acquainted with all the data collected through focus group discussion by revisiting the tape, read and re-read the transcribed data to make meaning out of them and record initial ideas. Generation of initial codes include the process in which the collected data was arranged in a meaningful and logical manner.

Searching for themes was initiated when the collating codes were developed into potential themes and all data relevant were gathered under each potential theme. Reviewing of themes involved the refinement of potential themes. Defining and naming themes means identifying the essence of what each theme is about (as well as the themes overall) and determining what aspect of the data each theme captures. Lastly, the step “writing the report” include the provision of a sufficient evidence of the themes within the data, that is, enough data extracted to demonstrate the prevalence of the theme.

The researchers prepared, organised, and transcribed data using Microsoft word from all focus group discussions (FGDs) and the transcribed data was sent to the independent co-coder for coding that followed the inductive thematic data analysis process until data saturation was evident. The themes and subthemes that were generated can be viewed in Table 1. The researchers and co-coder organised a meeting to discuss and agree on the themes and subthemes. The themes and sub-themes were described by the researcher, and participants were quoted. Moreover, the quotes were supported with literature during discussions. All data was managed using Microsoft word.

**Table 1:** The results of the study

Domains	Themes	Sub-themes
1 Advantages of CETU.	1.1 Benefit of CETU to staff.	**1.1.1** CETU is needed/necessary.
		**1.1.2** Staff development/ empowerment and Provision of learning opportunities.
	**1.2** Benefit of CETU to student nurses.	**1.2.1** Opportunity to integrate theory into practice.
		**1.2.2** Standardisation of procedures.
		**1.2.3** Strengthens supervision of student nurses.
	**1.3** Benefit of CETU to patient care.	**1.3.1** Upliftment of patient care.
		**1.3.2** Prevention of medico- legal risks.
2 Challenges of CETU.	**2.1** Inadequate resources for the establishment of CETU.	**2.1.1** Shortage of CETU staff.
		**2.1.2** Lack of CETU budget.
		**2.1.3** Lack of dedicated CETU structure.
		**2.1.4** Lack of CETU equipment.
3 Recommendations for CETU.	3.1 Suggestion to improve the establishment of CETU.	**3.1** Managerial support.
		**3.2** Provision of resources.

## Results

Nurses expressed different perceptions regarding the establishment of CETUs at their respective hospitals. Table 1 shows the results of this study, in which domains, themes, and sub-themes are illustrated. According to data comparison and analysis, excerpts were classified into three main studied domains as follows: advantages of CETU, challenges of CETU, and recommendations for CETU. Subsequently, a total of three main themes with eight sub-themes were determined for advantages of CETU, two themes with six sub-themes for challenges of CETU, and one theme with two sub-themes for recommendations for CETU respectively. Below is the description of the themes and sub-themes supported by quotes from FGDs.

### Advantages of CETU

CETU is viewed as an important unit in hospitals with the anticipated benefits that promote excellent clinical nursing skills to be implanted in nursing staff and student nurses, thus ensuring quality nursing care provision to healthcare users. The first domain was advantages of CETU in public hospitals. The advantages of establishing CETU at the hospitals are discussed under three main themes namely: benefit of CETU to staff, benefit of CETU to student nurses and benefit of CETU to patient care below:

#### Benefits of CETU to staff

The first theme focuses on the perceived benefits of CETU to nursing staff, and it consists of four subthemes namely, CETU is needed/necessary, staff development/empowerment, and the provision of learning opportunities. These sub-themes were discussed independently as follows:

- **CETU is needed/necessary**.

The subtheme that was first noted was the need to have CETU in hospitals of interest. Participants revealed that it is necessary to have CETU at their respective hospitals. Participants expressed their views as follows:

*In order for us to keep up with the current nursing trends*,* we need to have a training unit established to ensure that happens*. [Participant A, Hospital A]

*Now that CETU is a new programme under development*,* which according to my understanding teaches employees or the hospital staff the right way to do things. For example*,* if we are not aware of how to do vital signs*,* they are the ones who come here to teach us how to take vital signs so that we do them the right way*,* not the way we think they should be done. It is a good initiative because it will reinforce the correct way of doing things.* [Participant I, Hospital B]

*The presence of a CETU in our institution will help staff and students allocated in the units with the basic nursing procedures that CETU personnel demonstrate to them to ensure quality nursing care that will improve patient satisfaction.* [Participant K, Hospital C]

- **Staff development/empowerment and provision of learning opportunities**.

Staff development emerged as one of the anticipated benefits of CETU. Participants expressed that CETU plays a vital role in empowering nursing staff through the induction processes of the newly qualified nurse, and in-service training for nurses. Participants mentioned:

*CETU will benefit all of us*,* ranging from healthcare workers*,* student nurses*,* patients’ etcetera*,* in a sense that we going to be empowered with additional knowledge*,* and skills so that we can be more than competent and at the end of the day be able to perform our duties properly*,* professionally and to the satisfaction of our clients*. [Participant C, Hospital A]

*If there are any new updates*,* the CETU personnel keep us informed and updated on the current trends. Whenever they come to do their assessment*,* we keep on learning that things are no longer done like this*,* they have improved. CETU improves even staff*,* as our knowledge and skills increase through it*. [Participant K, Hospital C]

*CETU doesn’t only benefit student nurses*,* it also benefits permanent staff*,* for example*,* if you identify a certain knowledge gap in a unit*,* we can call the CETU personnel to assist. Remember nursing is all about continuous skills development*,* therefore it’s not only benefiting student nurses but also permanent personnel in the institutions*. [Participant H, Hospital B]

Participants surmised the presence of CETU will create a learning platform for staff at their hospitals. They uttered the following:

*If all of us in the hospital are up to date with the current trends it will also boost us in helping the students as well because if you don’t know*,* how are you going to help the students or transfer the skill you do not have? It will help us to sharpen our skills to have the boldness to help the students as well*. [Participant A, Hospital A]

*The CETU manager must only focus on the unit and nothing else but training and professional development of the nursing staff and the students*. [Participant S, Hospital D]

#### Benefits of CETU to student nurses

The second theme was the benefit of CETU towards the student nurses. It produced three subthemes namely: the opportunity to integrate theory into practice, standardisation of procedures, and strengthening students’ supervision. These subthemes were discussed individualistically as follows:

- **Opportunity to integrate theory into practice**.

The integration of theory into practice by student nurses was one of the identified subthemes. Participants were of the view that the establishment of CETU would provide student nurses the opportunity to integrate theory with practice. Participants articulated the following:

*CETU will also assist students during their clinical placement to be able to integrate theory with practice which eventually leads to quality nursing. Meaning they will get a better chance of putting what they have learned in a classroom environment into practice.* [Participant D, Hospital A]

*It will also assist in the integration between practical and theory. They won’t just meet practically during OSCE*,* this unit can assist them in preparing for OSCE*. [Participant G, Hospital B].

*The students will be supported by CETU personnel to incorporate the theory they learnt at school into practice when they are allocated to our institution*. [Participant K, Hospital C]

*CETU will also assist the students not only with the clinical aspect but also with their theoretical aspect*,* for example*,* if the students do not understand something in class*,* when they get to clinical placement*,* they can ask for clarity from the CETU.* [Participant O, Hospital D].

*CETU will also assist the students to have an insight as to how to approach certain exam questions relating to their real-life clinical practice experience and it will boost their confidence*. [Participant P, Hospital D]

- **Standardisation of procedures**.

The standardisation of procedures also emerged as a subtheme. Participants noted that the establishment of CETU will help in the standardisation of nursing procedures, they said:

*The CETU has been helpful to us because when there is an opening for in-service training*,* they inform us and coordinate the in-service training. The CETU manager also assists in the auditing of certain standards in the wards*,* and even the staff are informed that it is not only the operational managers who do the monitoring and evaluation. There has been a change since the CETU was established*,* as they also measure the quality of standards in the units*. [Participant J, Hospital C]

*I think that it is a very good idea since it is also going to help us in terms of research*,* for instance*,* if there are new protocols that we are not aware of*,* the CETU personnel will be the first person to be aware of the changes because they will be the first ones to be inducted about a certain change*,* and*,* in turn*,* they will transfer what has been said or taught to us as staff members*. [Participant E, Hospital B]

- **Strengthens supervision of student nurses**.

Supervision of student nurses was identified as the other subtheme. Participants emphasised that having CETU will assist in monitoring and supervising student nurses placed at their respective hospitals in a sense that the appointed CETU personnel will constantly check if students honour their placement. For example, if the student is placed in a medical ward, does the student complete the prescribed duration by placement at that particular ward or does the student self-place themselves according to preference. In addition, students’ absenteeism at clinical facilities will be documented, followed up, and action taken against such students, thus reducing deliberate absenteeism. Participants articulated as follows:

*The CETU members will be acting as police officers*,* making sure that nurses render services at the appropriate standard. Students won’t get a chance to roam and take shortcuts when it comes to nursing care because the CETU clinical nurse will be monitoring them.* [Participant O, Hospital D]

*Nurses do not have time to supervise students so if we have CETU it will assist in overseeing the students’ objectives when they are in clinical practice*. [Participant Q, Hospital D]

*We had a problem where the students were between the nursing education institution and the clinical area*,* and we did not have anyone to contact about such a student. Since the CETU manager is appointed in our institution*,* it is easier to do a follow-up of the students as she will call the relevant people to get clarity on the whereabouts of the students*. [Participant J, Hospital C]

*It will also assist in terms of discipline because several students do not follow the objectives that they come here for*. [Participant G, Hospital B]

*If there is a fully functional CETU in our hospital*,* student supervision will be intensified in the sense that students will report in the wards where they are placed as well as the CETU manager who will also take rounds to note students’ attendance*,* concerns*,* challenges*,* and progress*. [Participant F, Hospital B].

#### Benefits of CETU to patient care

The other theme that was identified was the benefits of CETU to patient care with the upliftment of patient care, and prevention of medico-legal risks as two subthemes. The subthemes are discussed below:

- **Upliftment of patient care**.

The first subtheme is the Upliftment of patient care. Participants believe that taking into consideration all other CETU benefits, patient care will be improved due to the sharpened skills brought by CETU to nurses and student nurses. Participants said:

*It also means that patient care will be improved because knowledgeable nursing leads to motivated nurses*. [Participant B, Hospital A]

*I believe there will be continuous education and training now and then*,* therefore nursing care for our patients will also improve to a larger extent*. [Participant C, Hospital A]

*It will help the staff and students allocated in the units with the basic nursing procedures that CETU personnel teach them to ensure quality nursing care*. [Participant K, Hospital C]

*The CETU personnel can remind me the correct way of suctioning a patient and revive that forgotten suctioning skill*,* I won’t be a hazard to the patients*,* and the patient’s hospital stay will be shortened*. [Participant A, Hospital A]

- **Prevention of medico-legal risks**.

*Prevention of medico-legal hazards was also identified as a subtheme. Participants believed that if CETU promoted the clinical skills of nurses and student nurses*,* the medico-legal hazard would be prevented or reduced. Participant explained*:

*It will also minimise the medico-legal hazards which usually occur in the workplace due to lack of knowledge or skill because now there will be staff that reinforce the correct way of doing things*. [Participant D, Hospital A]

*Having the necessary current knowledge and skills will reduce medico-legal hazards such as poor nursing intervention in the workplace*. [Participant C, Hospital A]

*If there is a CETU in place*,* the hospital will have fewer litigations because nursing staff will be trained and nursing standards will be followed*. [Participant Q, Hospital D]

### Challenges of CETU

The challenges of establishing CETU in the hospitals of interest was classified as a second domain. Despite the perceived benefit of CETU, there were also related challenges raised by participants that hindered the full establishment and functioning of CETU at their hospitals. Two themes emerged under the challenges domain, namely, inadequate resources for establishing CETU and Inadequate processes to facilitate the establishment of CETU and are as follows.

#### Inadequate resources for the establishment CETU

Inadequate resources for establishing CETU emerged as the first theme under this domain, and it has inadequate staff for CETU, lack of CETU budget, the absence of delegated structure for CETU, and lack of CETU equipment as subthemes. The subthemes were discussed as follows:

- **Shortage of CETU staff**.

Shortage of CETU staff emerged as the first subtheme. Participants raised their concerns regarding the absence of staff members dedicated specifically to CETU. Instead, the hospital management just delegates one staff member as a CETU manager. However, the participants believe that this is insufficient to run a fully functioning CETU. Participants uttered:

*There should be at least two members responsible for CETU to promote effective education. For example*,* if the current delegated CETU manager is on leave we should not have the challenge of saying the CETU manager is on leave for two weeks and the students are not attended to or there is no continuation of education*. [Participant E, Hospital B]

*The person heading the CETU office is there in the nursing staff structure meaning she is counted as nursing staff in the ward*,* but she is just delegated to CETU and not appointed as a permanent CETU personnel because of the passion she has for teaching students.* [Participant F, Hospital B]

*More staff members are needed to establish this CETU. When they are needed somewhere*,* there shouldn’t be this thing of shortage of staff*,* and you can’t go*. [Participant C, Hospital A]

*Currently*,* there is no appointment of staff members to work in this unit*. [Participant B, Hospital A]

- **Lack of CETU budget**.

The second subtheme that appeared was lack of a CETU budget. Participants highlighted lack of budget to ensure the smooth running of CETUs. Participants said:

*Remember if we say we establish this unit*,* we are talking about the budget. It is an issue already; our hospital is struggling when it comes to budget.* [Participant B, Hospital A]

*There must be a budget for CETU to function* [Participant R, Hospital D].

- **Lack of dedicated CETU structure**.

Lack of a dedicated CETU structure emerged as a third subtheme. Participants raised their concerns regarding lack of a suitable structure suitable for CETU. According to participants, the structures that are there are too small to be regarded as CETU structures, and in other cases, it is only the office of the delegated person for CETU. Participants said:

*I know the CETU managers have a place to work*,* an office kind of thing but the building is depleted. This tells you that CETU is not taken seriously because the building is falling*,* and it needs total restructuring.* [Participant M, Hospital C]

*No*,* there is no structure or classroom in place where CETU is running. What I know is that at the other hospital*,* there is a classroom that they use for teaching. A structure is needed for CETU to be established*. [Participant S, Hospital D]

*Honestly speaking*,* I only know the office*,* there is no specific venue or place for training*,* and it is only the office*. [Participant I, Hospital B]

- **Lack of CETU equipment**.

The fourth subtheme aroused is lack of CETU equipment. Participants commented that there is no equipment for CETU such as simulators and non-consumables. However, in some hospitals such as Hospital A, there are simulators, but they are not enough and in poor condition. Participants uttered:

*Unfortunately*,* we have a shortage of consumables in our hospital which makes it difficult for one to demonstrate. It is like you are just theorising*,* you are repeating what was taught in class*,* but we need to see it in life*. [Participant A, Hospital A]

*No*,* it does not have the equipment. The equipment has been ordered; we are waiting for it.* [Participant N, Hospital C]

### Recommendations for CETU

The recommendations that can be done to fully establish CETU were classified as a third domain. These recommendations are discussed further under one theme namely, suggestions to improve the establishment of CETU. This theme is discussed in detail below:

#### Suggestions to improve the establishment of CETU

This theme focuses on the suggestions made by the participants. Participants think these suggestions can help in improving the process of the establishment of CETU in the public hospitals of the North-West Province, South Africa. The theme encompasses two subthemes which are as follows: managerial support, and establishment of the CETU structure with equipment. The subthemes are respectively discussed as follows:

- **Managerial support**.

Management in support of CETU emerged as one of the subthemes. Participants suggested that the management of the hospitals should show satisfactory support to the full establishment of this unit so that it can function effectively as expected. Furthermore, other participants added that political will is also needed to fast-track the establishment of the unit. In addition to the managerial support and political will participants proposed that the presence of this unit must be communicated at all levels so that all the healthcare providers can be aware of its existence and that they can recognise and support the unit. Participants made the following suggestions:

*The one recommendation is that CETU is the custodian of nursing*,* and the nursing service manager is the one who is supposed to present the nursing operational plan including this unit. If nothing is happening at that level*,* we should not expect anything to happen down here. It is her role when they draft an operational plan for the financial year to include this section so that it can be fully functional*. [Participant B, Hospital A]

*The other thing that I recommend is that we should get a political-will that support the idea of CETU*,* because our managers can have the buy-in*,* but if there is no political will that drives this idea*,* we will take a longer time to can fully establish CETU*,* since politics can influence managers to accelerate the process of CETU.* [Participant C, Hospital A]

- **Provision of resources**.

Provision of adequate resources channelled toward the establishment of CETU emerged as one of the subthemes. Participants suggested that having sufficient resources for CETU could enable it to function efficiently. For instance, the provision of a budget so that the unit can be constructed, equipment such as simulators being purchased, and the appointment of dedicated CETU staff according to the recommended organogram in the Nursing Strategy. Participants articulated the following:

*The first thing that you must establish for CETU is a budget*,* therefore I recommend that there must be a proposed budget strictly for CETU*. [Participant R, Hospital D]

*For the unit to be established we need equipment for the training of student nurses and staff to be done effectively and efficiently. The budget for equipment should be approved so that necessary equipment can be ordered to ensure that clinical learning is more practical*. [Participant P, Hospital D]

*The National department has given us clear guidelines on the establishment of the CETU. According to the guidelines*,* there should be delegated staff*,* I think there should be five or six members who are supposed to be there. Others overlook specialty*,* others general programmes. This goes back to the issue of buy-in from the management*,* as they are in possession of the document and everything. The presentation was done but until they recognise that National is saying this should happen it becomes a problem*. [Participant B, Hospital A]

*I think a CETU must have its own staff because if you have to use nurses that are working in the units*,* they may not be able to fully function because on the other side they need to take care of patients. As much as we have other people helping in other units*,* there should be staff delegated strictly for CETU so that they can perform their duty not only when they are free but continuously*. [Participant A, Hospital A].

## Discussion

This study explored nurses’ perceptions regarding the establishment of CETUs in the Province of North-West Province, South Africa. The findings of the research study have being classified in three domains namely, advantages and benefits of CETU to staff, student nurses, and patient care, challenges which include inadequate resources for CETU establishment, and inadequate processes that facilitate the establishment of CETU. Lastly, recommendations with suggestions to improve the establishment of CETUs.

### The benefit of CETU to staff

The presence of CETU in clinical facilities is reported to have several benefits for staff. From the collected data, the benefit of CETU to staff appeared to be the first main theme and produced several subthemes. These subthemes include the need for CETU, the development and empowerment of staff, and the provision of learning opportunities. The study conducted by Hoshino et al., states that in-service education is implemented to improve the clinical/technical skills and critical thinking of new nurses, including new graduate nurses thus maintaining nursing competence [16]. This validates the researcher’s findings, which state that CETU is necessary.

In addition, the participants further reported that CETU provides staff with learning opportunities among others. The evidence from the findings of this study is supported by reviewed studies of Pedregosa et al. that reveal several potential benefits of dedicated educational units such as an increase in clinical problem identification, greater opportunities to practice clinical skills, and a significant improvement in patient communication, and a positive attitude toward the team process to mention a few [17]. As a results, it may be possible for staff members to be empowered.

### Benefit of CETU to student nurses

The benefit of CETU to student nurses surfaced as a second theme in this study with the integration of theory with practice and strengthen students’ supervision as subthemes. Jayasekara et al. found that clinical education is significant in developing nursing competencies and professional socialisation. Furthermore, clinical education also enhances students’ confidence in clinical practice, improving the sense of teamwork and organisational skills, as well as their readiness to become qualified professionals [18].

The integration of theory with practice was also reported as one benefits of CETU for student nurses. In simpler terms, this means student nurses can put what they have learnt in the classroom into practice. Subsequently, strengthening of students’ supervision also appeared as the other benefit. Bwanga and Chanda stated that clinical supervision allows students to safely apply theory into practice under the guidance of supervisors [19]. It is interesting to note that Bwanga and Chanda demonstrated the connection between clinical supervision and the integration of theory into practice [19].

### Benefits of CETU to patient care

An additional theme to surface was the benefit of CETU to patient care. The findings of the study also revealed the perceptions of professional nurses that stipulate that CETUs play a vital role in uplifting patient care and developing nursing skills that keep up with current health trends that enable nurses to perform their duties to the satisfaction of patients. According to Karaca and Durna, patient satisfaction is the most important indicator of quality of care, and it is considered an outcome of healthcare services [20].

It has commonly been assumed that, if the patient is satisfied it is an indication that the service received was of good quality. Equally, the increased competency among nursing staff and students has also been reported to result in the prevention of medico-legal risks. This assumption is supported by the study of Escudero et al. which states that the nursing staff that has the opportunity to practise skills and work through complex situations before facing the real environment is more likely to achieve patients’ safety and prevent the incidence of errors [21].

### Inadequate resources for the establishment of CETU

One other theme that emerged was the lack of resources necessary to guarantee the establishment of CETU. These resources include a structure specifically designed for CETU, equipment, staff, and most importantly, budget for CETU. In this study, it was established that in all hospitals, there is no structure with equipment dedicated to CETU, there are only offices where the CETU managers are stationed.

The study conducted by Bvumbwe and Mtshali, brings to light the fact that a shortage of teaching and learning resources results in inadequate productive capacity of training of nurses [22]. This projects the fact that inadequate resource for CETU has a detrimental effect on the training of staff and student nurses. In light of the fact that each hospital has only one designated CETU manager, the study confirmed that there is a shortage of staff members specifically for CETU. However, according to Lopez et al., the shortage of nurses has become a global phenomenon. Therefore, it may be concluded that lack of CETU practitioners in this study is negatively impacted by the overall nurse-shortage [23].

Among other things, it was reported that CETU has no budget, and it was believed to be a significant contributory factor to the lack of other resources at a broader scale. A study conducted by Spence et al., states that funding of clinical education is one of the costliest components, yet the most important variable in implementing such clinical education [1]. These contribute to our knowledge that operational challenges arise in the absence of financial support.

### Suggestions to improve the establishment of CETU

The last theme that emerged from the findings of this study was suggestions to improve the establishment of CETU. These suggestions were described by participants with the aim of assisting CETUs to be fully established at their respective hospitals. According to the study’s findings, guidelines outlining how CETUs should be established must be adhered to. More specifically, to guarantee the success of CETU establishment, these guidelines, which fall under the clinical teaching model, are found in the nursing strategy of 2012/2013–2016/2017.

These guidelines state that among other things staff establishment plan, an organogram, and a CETU structure with equipment must be realized [3]. Participants raised concerns that the management does not provide sufficient support for the establishment of this unit. Consequently, the participants implored the hospital management to provide sufficient support. For example, include CETU in their operational plans so that it becomes recognized and receives funds as an independent unit.

Spence et al., reaffirm that in order to guarantee the sustainability of clinical education, cooperative development supported by sufficient funding is required [1]. Therefore, these funds will be used to hire more staff and build a structure with equipment for CETU. Moreover, participants stressed that the notion that CETU needs political influence so that the establishment process can be accelerated. The suggestion of political influence is corroborated by Cao et al., who state that government policies play a vital role in driving important programmes along with administrative orders that can be imposed without any significant opposition [24].

### Limitations

This research study explored and described perceptions of professional nurses regarding the establishment of Clinical Education and Training Units in public hospitals in the North-West Province and the findings proposed implications for nursing education and training, practice, and research thereof. There are certain limitations of this study. Firstly, the number of participants per FDG was lower than expected mainly because of the unavailability of participants which may affect the precision of the study. Still, the researcher is of the opinion that the number of participants per FDG did not affect the practical significance of the study findings because FGDs lasted longer and data was collected until data saturation were reached. Secondly, the study was only conducted in four public hospitals in the North-West Province of South Africa. This denotes that the findings and results of this study are contextualised to the hospital settings of this province and cannot be generalized to public hospitals of other provinces. However, these findings can be applied in other studies.

## Implications of the study findings

### Nursing education and training

The findings of the study may contribute to fast-tracking a full establishment of CETUs at public hospitals of the NWP as the findings gleaned a wide range of views and ideas concerning CETU. Furthermore, the findings of this study suggest that student nurses might be able to integrate theory with practice effectively because there will be specific, fully functional units at hospitals that deal precisely with their clinical education and training. Moreover, nurse educators might be able to work together with professional nurses and clinical educators to promoting teamwork and student nurses might easily capture the practical aspects of their training because of good working relationships. Furthermore, professional nurses might be redirected to their role relating to CETU - for example, supervision and mentoring of student nurses.

### Nursing practice

The findings of this research study is important to the nursing practice in a sense that it may advance nursing care and improve patient outcomes. For example, when theory is integrated with practice, it will contribute to the nurses’ abilities to utilize evidence to increase understanding, stage strong arguments and make rational decisions. Subsequently, the clinical guidance and mentoring of student nurses will close the gap of the possible occurrence of adverse events that can predispose to an increase in mortality and morbidity rates from clinical mistakes and omissions that the student nurses do when there is no comprehensive guidance from professional nurses and clinical educators. On the other hand, student nurses will have more support from both clinical nurse educators and professional nurses in the wards.

### Nursing research

The study findings may add value to the repository of knowledge in nursing research by adding literature is what was already known about CETU. Moreover, other researchers may also utilise the information from the findings of this study to improve their studies.

## Conclusion

The main aim of this study was to explore and describe nurses’ perceptions regarding the establishment of Clinical Education and Training Units (CETUs) in public hospitals of the North-West Province, South Africa. The results and findings of the study reveal that there are anticipated benefits of establishing CETU in hospitals. Despite the anticipated benefits, related challenges were also identified. However, participants in this study suggested ways in which this unit can be fully established to function as expected. Most importantly, the researcher recommends that the well-known guideline of establishing CETU as publicised in the Nursing Strategy 2012/13–2016/17 of the National Department of Health must be utilised for direction. These guidelines were formulated specifically to ensure success in the establishment of these CETUs, and hence they are regarded as the cornerstone of CETUs.

## Data Availability

Data supporting the findings of this study can be requested from the authors concerned. Data is not available to the public due to privacy and ethical restrictions.

## Abbreviations

ACMC: Association of Canadian Medical Colleges
CEOs: Chief Executive Committee
CETUs: Clinical Education and Training Units
CTD: Clinical Teaching Department
CTUs: Clinical Teaching Units
DoH: Department of Health
FGDs: Focus group discussions
HREC: Health Research Ethic Committee
MTT: Ministerial Task Team
NDoH: National Department of Health
NSP: National Strategic Plan
NEIs: Nursing Education Institutions
NWP: North-West Province
NWU: North-West University
PHC: Primary Health Care
POPI: Protection of Private Information Act
SA: South Africa
SANC: South African Nursing Council
